# Treatment of NASH with Antioxidant Therapy: Beneficial Effect of Red Cabbage on Type 2 Diabetic Rats

**DOI:** 10.1155/2018/7019573

**Published:** 2018-09-30

**Authors:** Stéphanie Dal, Remmelt Van der Werf, Catherine Walter, William Bietiger, Elodie Seyfritz, Carole Mura, Claude Peronet, Julie Legrandois, Dalal Werner, Said Ennahar, Fabien Digel, Maillard-Pedracini Elisa, Michel Pinget, Nathalie Jeandidier, Eric Marchioni, Séverine Sigrist

**Affiliations:** ^1^Université de Strasbourg, Centre Européen d'Etude du Diabète, DIATHEC EA 7294, Fédération de Médecine Translationnelle de Strasbourg, F-67000 Strasbourg, France; ^2^Aérial, Illkirch, France; ^3^Equipe de Chimie Analytique des Molécules BioActives, IPHC-LC4, UMR 7178, Faculté de Pharmacie, Ilkirch, France; ^4^Interprofession des Fruits et Légumes d'Alsace (IFLA), Sainte Croix en Plaine, France; ^5^Structure d'Endocrinologie, Diabète, Nutrition et Addictologie, Pôle NUDE, Hôpitaux Universitaires de Strasbourg (HUS), 67000 Strasbourg, France

## Abstract

**Aims:**

Oxidative stress (OS) plays a major role in type 2 diabetes and its vascular and hepatic complications, and novel therapeutic approaches include natural antioxidants. Our previous chemical and biological studies demonstrated the antioxidant activities of red cabbage (RC), and here, we aimed to determine the *in vivo* effects of 2-month long RC consumption using a high-fat/high-fructose model of diabetic rats.

**Results:**

This vegetable, associated with lifestyle measurement, was shown to decrease OS and increase vascular endothelial NO synthase expression, ensuring vascular homeostasis. In the liver, RC consumption decreased OS by inhibiting p22phox expression and Nrf2 degradation and increasing catalase activity. It inhibited the activation of SREBP (1c, 2), ChREBP, NF-*κ*B, ERK1/2, PPAR*γ*, and GS and SIRT1 decrease, as observed in diabetic rats.

**Conclusion/innovation:**

RC consumption led to metabolic profile improvement, together with hepatic function improvements. Although lifestyle changes are not sufficient to prevent diabetic complications, enrichment with RC avoids progression hepatic complications. This antioxidant strategy using RC does not only able to increase antioxidant defense, such as classical antioxidant, but also able to assure a metabolic and energetic balance to reverse complications. Whereas traditional medical therapy failed to reverse NASH in diabetic patients, consumption of RC should be a natural therapy to treat it.

## 1. Introduction

In the last World Health Day organised by World Health Organization in 2016, it was reported that diabetes represents one of the main killers in the world characterised by an alarming explosion. In 2015, 415 million people worldwide were reported to have diabetes (1/11) and estimated at 642 million (1/10) in 2040, while 1/3 is expected to be overweight and 1/10 obese [1]. Key actions suggested to individuals are to avoid excessive weight gain, check blood glucose, follow medical advice, be physically active, and most importantly eat healthy [1], with the main objective of decreasing hyperglycaemia and avoiding diabetic complications.

Physiopathology of diabetes includes multiple disturbances, such as hyperglycaemia, glucose fluctuations, hyperinsulinaemia, hyperlipidaemia, and inflammation, all leading to the formation of reactive oxygen species (ROS) and exacerbation of oxidative stress (OS). OS is due to an imbalance between endogenous antioxidants (enzymes, vitamins, and proteins) and prooxidants (UV, alcohol, smoking, and endogenous enzymes) [2]. Many epidemiological and clinical studies strongly support ROS involvement in the genesis and evolution of chronic diseases, including diabetes and its complications [3]. Vascular complications are an important pathological issue leading to the further functional deterioration of several organs and caused microangiopathy and macroangiopathy [3]. Endothelial dysfunction, the loss of the balance vasodilator/vasoconstrictor factors, has been associated with type 2 diabetes (T2D) in several regions of the vasculature in animals and humans [4]. Due to defects in nitric oxide- (NO-) mediated relaxation [4], endothelial dysfunction has been associated with diabetic complications and recently with cardiovascular and all-cause mortality in diabetic patients [5]. Mechanisms underlying these processes are complex, and current research focuses on their aetiology, but OS is common for all [4, 6]. We have recently demonstrated in a T2D-model that OS is closely associated with vascular dysfunction and with the initial step and the progression of steatosis, especially in nonalcoholic steatohepatitis (NASH) [7]. Therefore, OS plays an important role in both cardiovascular [2] and liver complications [8] associated with diabetes.

The prevalence of nonalcoholic fatty liver disease (NAFLD) constantly increases; it represents the most common chronic liver diseases worldwide affecting up to 30% of the adult population and closely linked to obesity and diabetes development. It was reported that 20% of obese individuals present NASH compared with 3% of the nonobese whereas NAFLD affects 70–80% of obese and diabetic individuals [9]. Some of the most important issues in the current research are NASH pathogenesis and its underlying mechanisms. A growing body of evidence demonstrated that the primary abnormality involves metabolic disturbances, but the pathogenesis of NAFLD is multifactorial and includes lipid metabolism alterations, accumulation of triglycerides, inflammation, and OS, together with multiple closely related pathways [8]. Several studies reported an association between NAFLD and cardiovascular disease-related complications [10], and we have previously demonstrated that OS is involved in the induction of hepatic and peripheral vascular alterations in a T2D model [7]. However, no approved NASH therapies are currently available [10], highlighting the urgent need to develop effective therapeutic strategies for this condition.

The use of natural bioactive compounds may present a novel promising approach. Biological activities and beneficial properties of dietary polyphenols have been reported such as antioxidant, anti-inflammatory, antimicrobial, and immunomodulatory properties, in addition to protect *β*-cells from OS-induced loss of viability *in vitro* [11] and *in vivo* [12] and endothelial cells from OS-associated aging [13]. Moreover, preclinical and phase II clinical trials recently investigated natural therapy efficacy on hepatic signalling pathways [14], and several reports highlighted an inverse correlation between cardiometabolic mortality/morbidity risk and the consumption of polyphenol-rich products in animal diabetic models and human [6], especially fruits and vegetable consumption and reduction of diabetes [15, 16]. However, although the WHO estimates that the adequate consumption of fruits and vegetables may help save 1.7 million lives annually, the inadequate food consumption causes nearly 19% of gastrointestinal cancers, 31% of ischemic heart disease cases, and 11% stroke cases [17].

Our recent biological and chemical studies are aimed at studying polyphenolic composition of fruit and vegetable extracts and comparing their antioxidant properties. Using high-performance liquid chromatography (HPLC) coupled to the detection of free radical scavenging, we identified polyphenolic profile of red cabbage (RC) (*Brassica oleracea*) [18] and demonstrated its antioxidant activities. RC is rich in anthocyanin [19], one of the subclasses of phenolic phytochemicals, in the form of glycoside while anthocyanidin is known as the aglycone. Many studies have examined the effects of antioxidants on different aspects of diabetes, such as glucose metabolism, lipid profiles, or OS markers. However, no previous study has simultaneously examined multiple parameters in a diabetes model established by feeding healthy young rats with high-fat/high-fructose diet (HFHF) [7]. We aimed to assess the effects of chronic RC consumption in the T2D model and NAFLD complications. Here, the rats were administered HFHF for 2 months to induce diabetes, and afterward, one group was fed RC in addition to HFHF for the additional 2 months, while in the second group, HFHF was replaced by normal diet (ND) enriched with RC. The effects of these treatments on metabolic, oxidative, and inflammatory parameters and on the vessel and hepatic functions were assessed and compared with those of the rats fed ND for 4 months.

## 2. Materials and Methods

The study was performed in accordance with the “Guide for the Care and Use of Laboratory Animals” published by the National Institutes of Health (NIH publication no. 85-23, revised 1996) and approved by the regional committee (CREMEAS; approval AL/65/72/02/13).

### 2.1. Experimental Protocols

After 2 months of Western RD diet (Special Diets Services, Saint Gratien, France) plus 25% of fructose in water (HFHF) to induce T2D [7] on 8 weeks male Wistar rats (Depré, Saint Doulchard, France), HFHF rats (528 ± 7.2 g; 1.31 ± 0.05 g/L fasting glycaemia) were randomly divided into four groups for two more months: HFHF or HFHF plus red cabbage (RC) (HFHF/HFHFRC) represented “nutraceutical approach”; changed to normal diet (SAFE, Augy, France), plus water (HFHF/ND), or ND plus RC (HFHF/NDRC) represented “lifestyle measures,” in comparison to ND rats (494 ± 9.9 g; 0.97 ± 0.03 g/L). RC (var. Lectro) was given by the Interprofession des Fruits et Légumes d'Alsace (IFLA, Ste-Croix-en-Plaine, France), frozen after picking and lyophilised by the Centre d'Étude et de Valorisation des Algues (CEVA, Pleubian, France), crushed by Technopoudre (Ancenis, France), and incorporated in both diets at 10% by SDS (sodium dodecyl sulfate) for oral intake (Supplementary Table S1). Six rats were sacrificed at each time as previously described [7].

### 2.2. Plasmatic Metabolic and Oxidative Parameters

All the procedures to determine fasting glucose, c-peptide, insulin resistance, leptin, triglycerides, FFAs, adiponectin, *γ*-GT, total antioxidant capacity (TAOC), TBARS, and catalase and SOD activities are described in Supplementary Materials [7].

### 2.3. Vascular Analyses

The NO-mediated component of relaxation was determined in the main superior mesenteric artery rings suspended in organ baths to determine the changes in isometric tension [20] and described in Supplementary Materials. Vascular oxidative fluorescent dye dihydroethidine (DHE) was used to evaluate in situ formation of ROS [20]. eNOS and nitrotyrosine expressions were determined using immunohistochemical staining, described in Supplementary Materials.

### 2.4. Liver Analyses

All the procedures to determine the degree of hepatic histological changes were described above [7, 20]. Briefly, the degree of hepatic histological changes was assessed on 10 *μ*m cryosections fixed with 4% paraformaldehyde by eosin/hematoxylin coloration and Oil Red O staining (steatosis). Steatosis was evaluated according to the standard Kleiner Classification. In situ hepatic inflammation was on 10 *μ*m cryosections fixed and incubated with rabbit anti-Iba-1 (rat, 1 : 1000, Wako Chemicals GmbH, Germany). Hepatic oxidative stress was performed with a dihydroethidine (DHE) on unfixed 10 *μ*m thick sections that were treated with DHE (2.5 *μ*M) and incubated in a light-protected humidified chamber at 37°C for 30 min. Extraction and quantification of triglyceride (Abcam) and cholesterol (Cholesterol RTU™, Biomérieux) were performed on a piece of fresh liver (100 mg) according to the manufacturer's instructions. Extraction and quantification of glycogen content were also performed on a piece of fresh liver (100 mg) according to the manufacturer's instructions and expressed as glycogen/mg of liver.

Immunoblotting of target proteins was made through a traditional Western blot technic [21], on total protein extraction of the liver (80 *μ*g). SOD and catalase activities were determined on liver tissue supernatants [21]. All were presented in Supplementary Materials.

### 2.5. Statistical Analyses

Values are expressed as means ± standard error of mean (SEM), and *n* indicates the number of rats per group. Statistical analysis was performed with Student's *t*-test for unpaired data or analysis of variance (ANOVA) followed by Tukey's least significant difference (LSD) test after normality test validation, where appropriate (Statistica® version 12, StatSoft, France). *p* values < 0.05 were considered statistically significant (^∗^ vs. ND; ^$^ vs. HFHF; and ^#^ vs. HFHF/ND).

## 3. Results

### 3.1. RC Ameliorates Plasmatic Metabolic Disorders Associated with HFHF-Induced Diabetes

**Figure 1:**
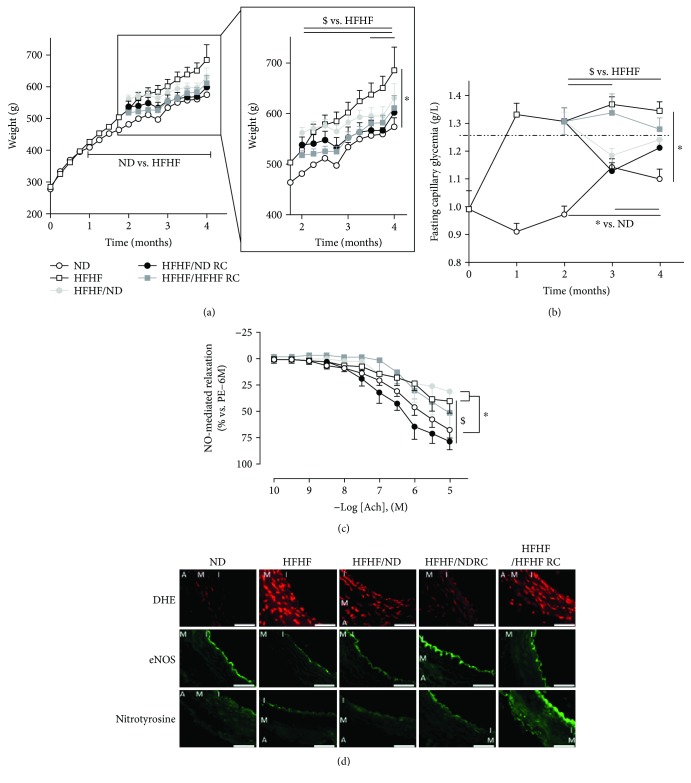
Weight, fasting glycaemia, and vascular function. (a) Weight and (b) fasting glycaemia levels during the study. (c) NO-mediated relaxation in mesenteric artery. (d) Characterisation of endothelial dysfunction through OS level analysis by dihydroethidine fluorescent probe (DHE), eNOS expression, nitrotyrosine formation. A: adventice, M: media, I: intima; bar scale = 50 *μ*m. All results are presented as mean ± SEM obtained in six experiments. ∗ significant difference vs. ND; $ vs. HFHF.

As previously described [7], HFHF induced obesity characterised by higher body weight, increase in BMI, abdominal circumference, and abdominal fat, compared with ND. HFHF/NDRC and HFHF/HFHFRC significantly limited this body weight gain (*p* < 0.05) with a BMI and abdominal fat levels in HFHF/NDRC rats similar to ND rats (Table 1, Figure 1(a)).

Furthermore, HFHF induced changes in metabolic control and diabetes development. HFHF rats presented fasting glycaemia higher than 1.26 g/L from 1 month after treatment onward (1.31 ± 0.04 g/L vs. 0.93 ± 0.05 g/L, data not shown), glucose intolerance with a high area under the curve (AUC) after intraperitoneal glucose tolerance test (ipGTT), hyperinsulinaemia and insulin resistance with an increase of C-peptide levels, and HOMA-IR higher than that in the observed ND group. HFHF induced dyslipidaemia as well, characterised by increased triglyceride, total cholesterol and free fatty acid (FFA) levels, associated with hyperleptinaemia, and increased adiponectin secretion (Table 1, Figure 1(b)).

HFHF/HFHFRC was shown to have no effects on hyperglycaemia, slightly decreased HOMA-IR, despite C-peptide decrease, improved dyslipidaemia (normalises FFA and decreases triglyceride, without affecting cholesterol), decreased leptin and adiponectin levels, and normalised glucose tolerance. HFHF/NDRC stabilized the fasting glycaemia to levels under 1.26 g/L, normalised dyslipidaemia and glucose tolerance, with the AUC after ipGTT and C-peptide comparable to ND rats, and decreased leptin and adiponectin (Table 1, Figure 1(b)).

### 3.2. RC Reduces Plasma OS Parameters by Increasing Total Antioxidant Capacity

HFHF led to increased plasma OS levels, which is characterised by an increase in TBARS and SOD activity and a decrease in catalase activity associated with an increase of vascular oxidative stress (Table 2).

Introduction of lifestyle measurement HFHF/ND significantly reduced total oxidative stress (TBARS, *p* < 0.01). Addition of CR did not improve this reduction but induced a slight increase of plasma total antioxidant capacity (*p* < 0.05) associated with an increase of catalase activity (*p* < 0.05). Finally, nutraceutical approach alone (HFHF/HFHFRC) led only to decreased TBARS (*p* < 0.05) (Table 2).

### 3.3. RC Reduces HFHF-Induced Endothelial Dysfunction by Decreasing Vascular OS Levels and Promotes NO Bioavailability by Increasing eNOS Expression

Endothelium plays a key role in vascular homeostasis by regulating the balance between relaxing and contracting factors. The addition of acetylcholine leads to NO-mediated concentration-dependent relaxation in mesenteric artery rings with endothelium of ND group (at 10^−5^ M, 67 ± 7%). However, this relaxation was reduced twofold in the HFHF group (40 ± 11%) (Figure 1(c)). Moreover, HFHF induced a twofold increase of ROS levels and a decrease of eNOS expression in comparison to the ND group, without the formation of peroxynitrites, as assessed by nitrotyrosine immunofluorescence (quantification of vascular oxidative stress Table 2, Figure 1(d)).

HFHF/HFHFRC presented also blunted NO relaxation (at 10^−5^ M, 50 ± 16%) associated to an increase of ROS and nitrotyrosine staining in arteries. But, eNOS expression was equal to the ND group. HFHF/NDRC showed more pronounced effects, restored the relaxation more than the administration of ND (78 ± 8%), increased eNOS expression without the formation of peroxynitrites, and decreased ROS across the entire vasculature (Table 2, Figures 1(c) and 1(d)).

### 3.4. RC Ameliorates HFHF-Induced NASH

**Figure 2:**
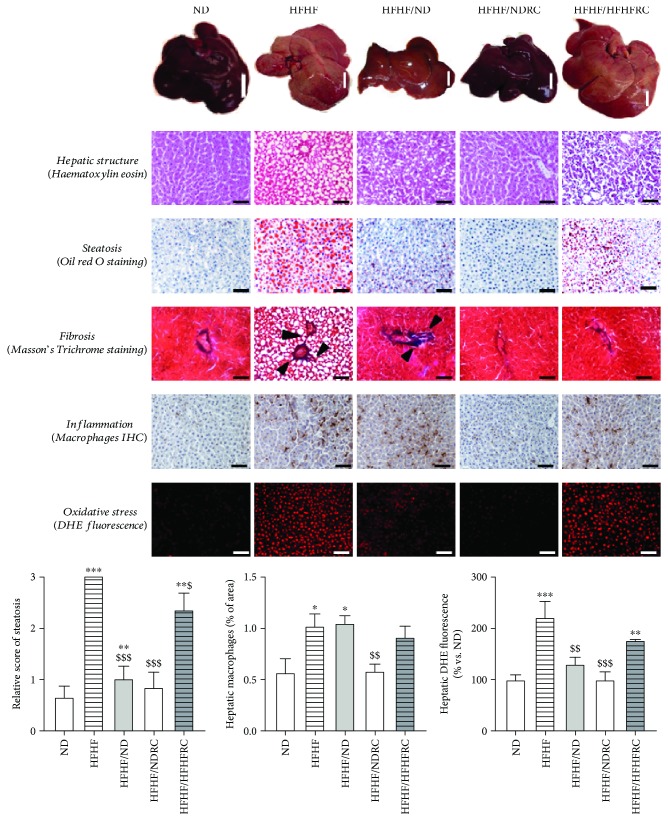
Hepatic complications. Severity of hepatic complications was visualised in the representative liver images (bar, 1.5 cm), haematoxylin/eosin, Oil-Red O and Masson's Trichrome stainings, macrophage immunohistochemical, and DHE fluorescence. Bar = 100 *μ*m. All results are presented as mean ± SEM of the results obtained in six experiments. ∗ significant difference vs. ND; $ vs. HFHF.

Histological analysis of hepatic tissue from ND rats showed that the cytoplasm of hepatocytes was homogeneously coloured pink, the nuclei were well defined by violet staining, and cells were organised radially around centro-lobular veins without fibrosis, resulting in a steatosis score of 0, according to Kleiner et al. [22]. However, HFHF liver showed vacuolar degeneration with ballooned vacuoles and many droplet accumulation with nuclei less visible and which were at the edge of the cytoplasm. These results suggested hepatic fat accumulation confirmed by elevenfold increase in hepatic triglyceride levels, together with fourfold increase in hepatic cholesterol contents (Table 2). Maximum hepatic steatosis score was observed in HFHF rats, unlike that in ND rats (3 ± 0 vs. 0.67 ± 0.21) (Figure 2). Hepatic glycogen contents were shown to decrease twofold following the administration of HFHF (data not shown), while the levels of glycogen synthase (GS) were involved in the conversion of glucose into its polymeric form glycogen and were shown to decrease more than twofold with HFHF (Table 2). Steatosis was associated with macrophage infiltration, and some collagen fibres started growing from the portal system as assessed by Masson's Trichrome staining (Figure 2). One of the crucial factors involved in the damaging of hepatocytes is OS [8], and, similar to the results obtained in our previous study [7], HFHF was shown to induce hepatic ROS levels (221.6 ± 31.33%) in comparison with those in the ND group (100 ± 7.75%) (Figure 2). This led to the development of hepatomegaly and hepatic dysfunction with an increase in *γ*-GT (Table 2), without affecting alanine aminotransferase (data not shown).

**Figure 3:**
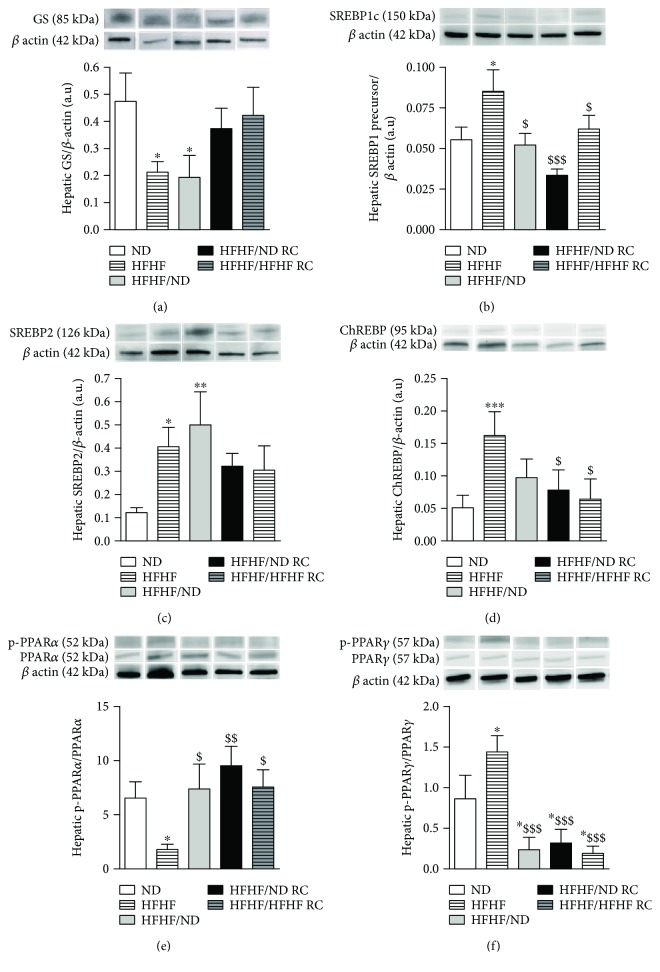
Hepatic glucose and lipid metabolism signalling pathways. Expression of (a) GS, (b) SREBP1c, (c) SREPB2, (d) ChREBP, and (e, f) PPAR*α* and PPAR*γ* and their phosphorylated form (p-). All results are presented as mean ± SEM of the results obtained in six experiments. ∗ represent significant difference vs. ND; $ vs. HFHF.

HFHF/HFHFRC was shown to lead to a slight amelioration of steatosis (2.33 ± 0.2), without beneficial effect on triglyceride content and hepatic dysfunction (high triglyceride and *γ*-GT). Furthermore, macrophage infiltration and OS levels did not change, since hepatic ROS levels were shown to be 176 ± 1.26%. However, HFHF/HFHFRC maintained glycogen content through maintaining the physiological levels of GS and decreasing cholesterol. No fibrosis was detected, despite the presence of hepatomegaly. Histological analysis of hepatic tissue from HFHF/NDRC rats showed that the hepatocyte cytoplasm regions were homogeneously stained, nuclei were well defined by violet staining, and cells were organised radially around centro-lobular veins without fibrosis, triglyceride accumulation, or cholesterol, resulting in a steatosis score of 0.5 ± 0.22. Glycogen storage and GS expression did not differ from those observed in the ND group, while the OS was shown to be completely abolished following the consumption of RC (97.07 ± 17.31%), together with macrophage infiltration, leading to improvement in hepatomegaly (Table 2, Figures 2 and 3(a)).

### 3.5. RC Promotes Oxidative Balance and Decrease Inflammation in the Liver

**Figure 4:**
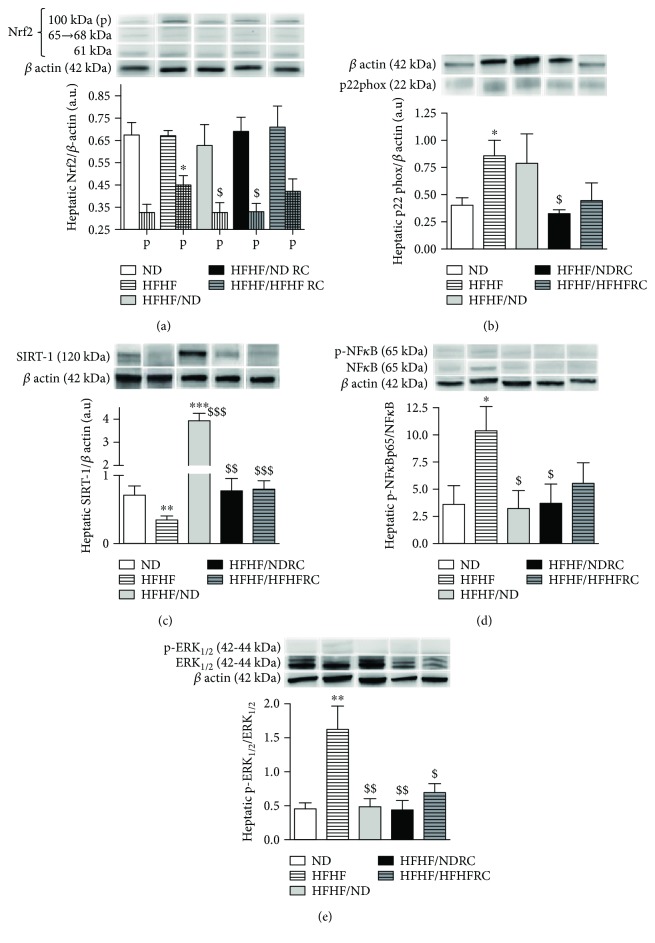
Hepatic oxidative stress markers. Expression of (a) Nrf2 and its polyubiquitinated form (P), (b) p22phox, (c) SIRT-1 (d), NF-*κ*B, (e) ERK1/2 and their phosphorylated form (p-). All results are presented as mean ± SEM obtained in six experiments. ∗ significant difference vs. ND; $ vs. HFHF.

Despite HFHF-induced hepatic ROS formation, the activity of SOD and catalase did not differ from that in the ND group. The level of the polyubiquitinated form of Nrf2, which regulates the expression of antioxidant and cytoprotective genes in response to the increased levels of electrophiles and OS [23], increased 1.5-fold following the administration of HFHF. Moreover, the expression of p22phox, NADPH oxidase (Nox) subunit and a major source of glucose-induced ROS production in the liver [21], was twofold increase (Table 2, Figures 4(a) and 4(b)). The degradation of Nrf2 and p22phox expression was inhibited only with HFHF/NDRC whereas HFHF/HFHFRC tended to decrease them (*p* = 0.06 vs. HFHF). Moreover, HFHF/NDRC increased catalase activity and HFHF/HFHFRC decreased it, all in accordance with the different effects of these two diets on hepatic ROS formation (Table 2, Figures 2 and 4(b)).

HFHF led to a twofold reduction of SIRT1 expression, which regulates glucose/lipid metabolism, OS, and inflammation, partially by physical interaction with the p65 subunit of NF-*κ*B [24], and increased threefold the levels of its phosphorylation leading to macrophage infiltration (Figure 2). HFHF led to a threefold increase in the phosphorylation of ERK1/2, where extracellular kinases were frequently activated by mitogens and growth factors [25] (Figures 4(c)–4(e)). HFHF/HFHFRC maintained SIRT1 and ERK1/2 expression levels at those observed in the ND group, and the phosphorylation of NF-*κ*B showed the tendency to decrease in comparison to the HFHF group (*p* = 0.078 vs. HFHF). However, HFHF/NDRC led to the stabilization of physiological levels of NF-*κ*B and (p)-NF-*κ*B and ERK1/2 (Figures 4(c)–4(e)).

### 3.6. RC Promotes Hepatic Metabolism Pathways

HFHF increased SREBP-1c and ChREBP, two major transcription factors implicated in liver lipogenesis [8], and SREBP2, the master regulator of intracellular cholesterol homeostasis [14]. Both RC treatments decreased SREBP-1c and ChREBP levels and assured an intermediate SREBP2 level (Figures 3(b)–3(d)).

HFHF induced the phosphorylation of PPAR*γ*, which plays a role in the process of lipid storage [14], while it led to a threefold decrease in PPAR*α* phosphorylation, which was suggested to be involved in the process of fat catabolism [14]. HFHF/HFHFRC or HFHF/NDRC led to a decrease in PPAR*γ* levels to the levels lower than those observed in ND rats, while maintaining normal PPAR*α* phosphorylation levels (Figures 3(e) and 3(f)).

**Figure 5:**
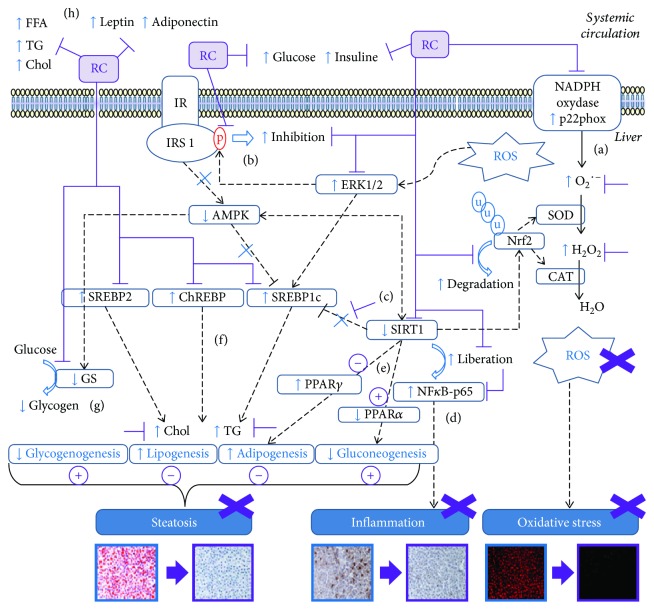
Beneficial effect of RC on hepatic glucose and lipid metabolism signalling pathways. RC prevents hepatic damages associated to NAFLD through different mechanisms: (a) promotes oxidative balance: ROS, prooxidant p22phox, Erk1/2, and Nrf2 degradation inhibitions; (b) improves insulin sensitivity: inhibits IRS1^Ser^-phosphorylation; (c) normalises SIRT1 levels and promotes its beneficial effects; (d) NF-κB and inflammatory pathway attenuation; (e) increases *β*-fatty acid oxidation and gluconeogenesis: PPAR*α* stimulation, PPAR*γ*, and adipogenesis inhibitions; (f) reduces de novo lipogenesis: maintains SREPB1c, ChREBP, and SREPB2 physiological levels, decreases hepatic triglycerides and cholesterol; (g) increases glycogenogenesis rate: GS expression normalisation; (h) ameliorates plasma adipokine and lipid contents. u: ubiquitin; ---: interpathway interactions; →: stimulatory activity; ┤: inhibitory activity; X: suppression.

Figure 5 presents signalling pathways of beneficial effects of RC on hepatic glucose and lipid metabolism.

## 4. Discussion

Many previous studies reported that antioxidants have protective effects against diabetes and its complications [6]. The ISA-FRUIT Project financed by the European Union in 2008 and the EPIC-Norfolk Study have demonstrated an inverse correlation between the consumption of “unprocessed” F&V and health outcomes, including obesity and diabetes [6]. F&V plays an important role in the redox status maintenance, regulating OS levels [6]. RC, an endemic Mediterranean region vegetable consumed as coleslaw, salad, or beverage, has antioxidant properties *in vitro* [19], cooked or uncooked (Supplementary Materials, Figure S1), and *in vivo* [26]. Anthocyanins, natural pigments present in dark-coloured F&V such as RC, have the strongest antioxidant properties among 150 examined flavonoids [27]. Therefore, we investigated the potential of RC consumption for the prevention of vasculature and liver complications associated with T2D using HFHF rats.

We demonstrated that RC administered together with HFHF or ND and HFHF/ND decreased weight, BMI, and abdominal circumference, which may indicate that this vegetable helps induce the use of fat and may induce lipid trafficking away from the abdomen reducing the severity of associated complications, mainly NAFLD and cardiovascular remodelling. Moreover, HFHF/NDRC was shown to be the only diet leading to the long-term normalisation of all metabolic parameters, such as hyperglycaemia, hyperinsulinaemia, hyperleptinaemia, dyslipidaemia, and hypercholesterolaemia, and to decrease insulin resistance.

Recent clinical intervention studies showed that anthocyanin decreases them [15, 16, 28] leading to 15% reduction in T2D risk [15]. In spite of the observed beneficial effects of HFHF/HFHFRC on the reduction of weight, it induced considerable hyperleptinaemia associated with the decrease in food intake, most likely due to the effects of leptin on satiety [29]. According to previous studies, anthocyanins have a suppressive role in the development of adipocyte hypertrophy and reduce hyperglycaemia in HF mice [30], and cyanidins induced leptin and adiponectin production in isolated adipocytes [31]. All these processes limit the accumulation of FFAs, as observed in HFHFRC rats. However, this hyperleptinaemia is induced only when RC is consumed in the presence of hypertrophied adipocytes, since HFHF/NDCR rats have physiological levels of leptin and adiponectin. RC was shown to reduce glycaemia and cholesterol [15, 32] leading to a reduction in diabetes and heart disease risk [15].

We showed that the consumption of RC with HFHF or ND and HFHF/ND led to the reduction in the plasmatic OS-related complications such as TBARS, by increasing SOD and normalising catalase activities. However, only HFHF/NDCR led to an increase in TAOC and inhibited OS pathway activation in vessels and the liver, which indicates that RC promotes oxidative homeostasis. In the mesenteric artery, HFHF/NDCR decreased HFHF-induced OS and increased eNOS expression leading to physiological NO-mediated relaxations. However, OS signalling was shown to persist in the vessels of HFHF/ND or HFHF/HFHFRC rats, which was not accompanied by alterations in eNOS expression but an increase peroxynitrite levels and a decrease in relaxations. RC was shown to promote vascular homeostasis, as demonstrated by the improved NO bioavailability. The blunted NO availability is believed to be the primary defect linking insulin resistance and endothelial dysfunction [4] and to be associated with OS [7]. This link between endothelial dysfunction, insulin resistance, and OS was also identified as a cause underlying cardiovascular and all-cause mortality in diabetic patients [5]. However, a recent extensive meta-analysis including 136,846 participants showed that adherence to the Mediterranean diet, rich in polyphenol-derived products, is associated with a 23% reduction in T2D risk confirming its beneficial effects on endothelial function and inflammation rates [33]. Beneficial effect of HFHF/NDCR may be explained by the long-term decrease in blood glucose, plasma FFA, and cholesterol levels. Hyperglycaemia, OS, and diabetic complications have been linked previously, since endothelial cells were shown to be permeable to glucose, and its metabolism leads to the generation of high quantities of superoxide anions [3, 6]. Moreover, the induction of hepatic OS, through the activation of NADPH oxidase pathway, leads to decrease in glycogen and GS levels [7, 34]. All pathological alterations were shown to be improved by HFHF/NDCR, which induced partial decrease in fasting glycaemia. RC, through the inhibition of superoxide accumulation and/or by the modulation of blood glucose levels, prevented disorders, as shown with anthocyanins [35]. Therefore, we demonstrated that only the simultaneous control of glycaemia and OS can help normalise endothelial function, as previously described in T1D patients, while early hyperglycaemia or long-lasting poor glycaemic control can result in the long-term endothelial dysfunction due to the metabolic memory [36]. Metabolic memory is the idea that early glycaemic environment is remembered in the target organs and thus diabetic vascular stresses persist after complete glucose normalisation. One of the principal mechanisms highlighted was OS [37, 38] that is why glycaemic and oxidative control were needed to assure a great endothelial function as shown with HFHF/NDRC.

Moreover, FFAs induced impaired fasting glucose levels, insulin resistance, increased inflammation, and OS leading to vascular dysfunction [7, 39]. All of these parameters were normalised only following the administration of RC and HFHF/ND.

Furthermore, we showed that HFHF/NDRC consumption decreases body weight/liver weight ratio and suppresses hepatic steatosis, fibrosis, inflammation, and OS complications. We observed that RC intake was able to normalise the expression of prooxidant enzymes such as p22phox, inhibited Nrf2 degradation, and prevented the impairment of enzymatic antioxidant leading to increased catalase activity and decreased ROS. Moreover, superoxide and H_2_O_2_ have been described to activate MAPK family cascade at MEK (MAPK/ERK kinase) and ERK1/2 levels [25] confirming our results highlighting an increase of ROS and ERK1/2, but not p38 kinase activity (data not shown). RC decreased ERK1/2 activation as shown *in vitro* with cyanidins [40]. While all these disorders were also reported in NAFLD patients [8] and fibrosis [41], traditional therapeutic approach using ND without RC failed to decrease p22phox expression and fibrosis. So, our results showed that RC adds to standard lifestyle measures promoting hepatic homeostasis, normalising hepatic oxidant pathway, and improving NASH symptoms.

During increased OS levels and MAPK activation, or in the presence of other diabetogenic factors, such as FFAs and hyperinsulinaemia, several serine/threonine kinases may be activated, IRS1 is phosphorylated, and protein degradation rates increase [42]. RC was shown to protect against the activation of these processes, but it was not able to prevent the inhibition of a downstream molecule, AMPK induced by HFHF and obesity (Supplementary Materials, Table S1). Therefore, RC consumption seems to preferentially affect the downstream pathway. However, certain OS-related defects in oxidative phosphorylation machinery and mitochondrial *β*-oxidation lead to the excessive accumulation of hepatic triglycerides and subsequent development of insulin resistance [43], and the results obtained here show that RC consumption and ND prevent insulin resistance development. The abnormal expression profiles of hepatic lipogenic transcription factors and enzymes induced by HFHF indicate that high-energy and high-carbohydrate diets, especially sugars, increase de novo lipogenesis in the liver and decrease insulin sensitivity, due to fructose lipogenic potential during liver metabolism [44]. Moreover, changes in adipocytokine secretion in fat tissue such as the production of leptin, cholesterol, and triglycerides, as shown in our study, lead to the development of proinflammatory and profibrotic states [8, 45, 46]. Adiponectin which generally negatively correlates with the body fat, fasting insulin, and oral glucose tolerance were increased in our HFHF model as observed in obese patients with liver dysfunction and insulin resistance [47]. The consumption of RC and ND helped in maintaining physiological levels of leptin and adiponectin which are associated with better hepatic environment.

In our model, hyperglycaemia led to the activation of ChREBP, which partially controls hepatic lipogenesis, while hyperinsulinaemia activates SREBP1c which regulates a different set of genes involved in hepatic lipogenesis. Additionally, SREBP2 levels were shown to be increased as well, and SREBP2 regulates intracellular cholesterol homeostasis and was recently associated with NASH [14]. A number of *in vitro* and *in vivo* studies investigating polyphenols such as anthocyanin showed that the expression of SREBP1c and its main targets is downregulated during lipogenesis [48]. In our study, RC consumption, together with ND or HFHF intake, allowed the normalisation of the levels of these lipogenic factors, followed by a decrease in hepatic triglyceride and cholesterol contents and steatosis.

Studies reported that SIRT1 is a key regulator of de novo lipogenesis, insulin sensitivity, mitochondrial fatty acid oxidation, and lipolysis [24]. It can affect Nrf2 expression and may protect liver against an injury, and we showed that RC is responsible for the normalisation of SIRT1, SREBP1, and PPAR*α* levels suggesting their potentially important role in RC-mediated protection against the development of NASH. Previously, it was reported that a decrease in SIRT1 expression inhibits SREBP1 and lipogenesis [49], decreases PPAR*α* levels which leads to the reduced use of fat as an energy source, and induces fat accumulation and hepatic steatosis [14, 49]. RC maintained the physiological levels of the components of these pathways, promoting their improved balance through lipogenesis, adipogenesis, and gluconeogenesis. As shown in HF mice, anthocyanin-rich juice supplementation stimulated PPAR*α* upregulation in parallel with the downregulation of *de novo* expression of lipogenic genes in the liver [50]. Simultaneously, PPAR*γ* levels were shown to be upregulated in HFHF rats with severe hepatic steatosis. PPAR*γ* plays an important role in the process of lipid storage, and it is involved in SREBP1 activation and SIRT1 inhibition leading to fat accumulation and *β*-fatty acid oxidation decrease. These results are in accordance with those obtained in two mouse models with the liver-specific gene deletions of *PPARγ*, ob/ob, and A-ZIP/F-1, where hepatic steatosis was shown to be markedly attenuated [51]. Finally, SIRT1 was shown to inhibit NF*κ*Bp65 and inflammation [24]. However, SIRT1 inhibition in HFHF rats may lead to an increase in NF-*κ*B phosphorylation and macrophage infiltration. RC and ND normalised SIRT1 levels, which improved glucose tolerance, enhanced systemic insulin sensitivity, and normalised the levels of tissue markers of inflammation [52].

If the notion of oxidative stress and antioxidant therapy were very controversial a fortnight ago [53] with chemical and cellular beneficial effect without impact on animals [54], results should stimulate, rather than discourage, important research in this field. Quideau et al. [55] and others reviewed the chemical perspective of polyphenol compounds, summarise the state of the art, and highlight the most significant advances. Increasing endogenous antioxidant levels may be a better approach to therapeutics and disease prevention than consuming large doses of “dietary antioxidants” [56] as showed with RC. Today, several studies in animal models and human subjects have demonstrated that phenols are bioavailable and exert a protective role against oxidative stress and free radical damage [6, 57]. Moreover, epidemiological studies suggest that consumption of fruits, vegetables, and plants [6] may be associated with a reduced risk of diabetes or have a protective effect [58].

Finally, our results are in accordance with the results of a recent report that analysed data obtained in three prospective cohort studies involving 200,000 USA men and women, which reported an inverse association between the consumption of anthocyanins/anthocyanin-rich foods and diabetes, while the strongest association was observed for cyanidins, one of the six most common anthocyanidins studied (cyanidin, delphinidin, malvidin, pelargonidin, peonidin, and petunidin) [16] present in RC. Antioxidants found in nature may represent promising therapeutics for the treatment of diabetes, or improvement of the complications associated with it. Moreover, two recent reviews summarised current and emerging therapeutic approaches to NAFLD treatment [14, 59] and highlighted the importance of novel targets, such as PPARs, Nrf2, SREBP2, and SIRT1, in the treatment of fat accumulation, inflammation, and OS. Lifestyle changes are certainly essential in the management of diabetes, but they seem to be insufficient. However, diet enrichment with rich sources of bioactive compounds, such as RC, would ensure optimum efficacy. We showed that RC can normalise the levels of many of these potential therapeutic targets, which supports the importance of its consumption as an adjuvant in the therapy of T2D patients with metabolic, vascular, and hepatic complications.

## Figures and Tables

**Table 1:** Anthropomorphic and metabolic characteristics of rats after 4 months of the administration of different diets.

Variables	ND	HFHF	HFHF/ND	HFHF/NDRC	HFHF/HFHFRC
*Physiological variables*					
Weight gain from 2 to 4 months (g)	63.4 ± 6.9	97.5 ± 4.60^∗^	13 ± 12.14^∗∗∗^^$$$^	15.67 ± 12.77^∗∗^^$$$^	64.58 ± 6.7^$^
Final body weight (g)	558 ± 15.3	628 ± 12.6^∗^	570 ± 13.4^$$^	533 ± 19.8^$$$^	574.25 ± 10.43^$^
Body mass index (BMI; g/cm^2^)	0.78 ± 0.003	0.86 ± 0.02^∗^	0.76 ± 0.02^$$^	0.77 ± 0.01^$^	0.83 ± 0.03
Abdominal circumference (cm)	22.14 ± 0.59	24.9 ± 0.78^∗^	22.08 ± 1.34^$^	22.50 ± 0.87^$^	23.08 ± 0.61
Abdominal fat (g) (% vs. total weight)	12.22 ± 1.74 2.17 ± 0.27	34.60 ± 1.38^∗∗∗^ 4.37 ± 0.96^∗∗∗^	18.33 ± 3.39^$$$^ 3.17 ± 0.49^$$$^	15.38 ± 2.81^$$$^ 2.84 ± 0.45^$$$^	21.65 ± 3.01^∗^^$$^ 2.55 ± 0.90^∗^^$$^
*Plasmatic metabolic variables*					
Fasting blood glucose (g/L)	1.10 ± 0.04	1.34 ± 0.04^∗∗∗^	1.24 ± 0.02^∗∗^^$^	1.21 ± 0.3^∗^^$$^	1.28 ± 0.04^∗∗∗^
Area under the curve during IpGTT	244.5 ± 18.3	343.2 ± 29.3^∗^	274.5 ± 16.22^$^	243.2 ± 10.32^$$$^	273.5 ± 13.47^$^
Plasma C-peptide (pmol/L) fasting 60 min post ipGTT	583 ± 80.47 2274 ± 283.9	1954 ± 266.7^∗∗^ 4398 ± 298.6^∗∗∗^	1179 ± 91.03 3167 ± 783.5^$$^	1081 ± 68.94 2479 ± 197.6^$$$^	1163 ± 172.6 2944 ± 334.7^$$^
Insulin resistance (HOMA-IR)	1.43 ± 0.15	4.84 ± 0.63^∗∗∗^	2.87 ± 0.23^∗∗∗^^$$$^	2.57 ± 0.15^∗∗∗^^$$$^	2.87 ± 0.44^∗∗∗^^$$^
Fasting plasma leptin (ng/mL)	13.23 ± 0.99	37.24 ± 5.39^∗∗∗^	15.88 ± 1.08^$$$^	14.98 ± 1.99^$$$^	27.48 ± 4.42^∗∗^^$^
Plasma triglycerides (*μ*mol/L)	805.2 ± 175.2	1523 ± 184.6^∗∗^	878 ± 199.7^$^	565.1 ± 96.7^$$^	1066 ± 282.6
Plasma adiponectin (ng/mL)	4184 ± 221	6005 ± 359^∗∗∗^	4439 ± 197^$$$^	4893 ± 279^$$^	5000 ± 134.8^∗^^$$^
Plasma free fatty acids (FFA; *μ*mol/L)	82.11 ± 10.64	234.4 ± 29.39^∗∗∗^	155.3 ± 14.12^∗^^$$^	11.95 ± 4.13^∗^^$$$^	55.27 ± 24.84^$$$^
Plasma total cholesterol (mmol/L)	1.37 ± 0.12	2.21 ± 0.30^∗∗^	1.71 ± 0.25	1.58 ± 0.08^$^	2.485 ± 0.19^∗∗∗^

**Table 2:** Oxidative stress levels and rat hepatic functions at 4 months after the administration of different diets.

Variables	ND	HFHF	HFHF/ND	HFHF/NDRC	HFHF/HFHFRC
*Plasma oxidative stress parameters*					
Plasma total antioxidant capacity (mM trolox equivalent)	5.75 ± 0.18	5.75 ± 1.167	5.99 ± 0.131	6.43 ± 0.35^∗^^$^	6.18 ± 0.26
Plasma lipid peroxide, TBARS (*μ*M malondialdehyde)	35 ± 1.14	66.98 ± 9.57^∗∗^	34.7 ± 0.925^$$^	34.67 ± 3.097^$$^	41.93 ± 6.02^$^
Catalase activity (*μ*mol/L) SOD activity (% inhibition)	0.0516 ± 0.03 57.52 ± 5.83	0.022 ± 0.004^∗^ 71.43 ± 4.22^∗^	0.039 ± 0.011 81.11 ± 1.98^∗∗^	0.044 ± 0.010$ 80.9 ± 1.36^∗∗^	0.043 ± 0.006 81.11 ± 2.49^∗∗^
*Vascular oxidative stress*					
DHE fluorescence (% vs. ND)	100 ± 18.5	183.3 ± 7.1^∗∗∗^	160.9 ± 17.1^∗∗^	124.2 ± 7.4$$	200.2 ± 17.1^∗∗∗^
eNOS fluorescence (% vs. ND)	100 ± 10.7	53.3 ± 7.2^∗∗∗^	78 ± 3.01^$^	131.5 ± 10^∗^^$$$^	111.05 ± 18.05^$^
Nitrotyrosine fluorescence (% vs. ND)	100 ± 8.3	113.5 ± 15	107.4 ± 7.8	100.3 ± 11.2	162.1 ± 31.3^∗^
*Hepatic oxidative stress*					
Catalase activity (g/mL) SOD activity (% inhibition)	0.50 ± 0.01 99.9 ± 0.39	0.49 ± 0.01 105.4 ± 5.09	0.53 ± 0.012^∗^^$$^ 103.3 ± 1.84	0.53 ± 0.006^∗^^$$^ 99.75 ± 1.33	0.47 ± 0.003^∗^ 103.3 ± 0.78
*Hepatic function*					
Liver weight (g) (% vs. rat weight)	17.12 ± 0.45 3.07 ± 0.03	22.28 ± 2.01^∗∗^ 3.50 ± 0.28	17.83 ± 0.78^$^ 3.13 ± 0.12	17.53 ± 1.12^$^ 3.28 ± 0.13	21.37 ± 1.25^∗^ 3.71 ± 0.16^∗∗^^#^
Triglycerides (nmol/mg of liver)	1.8 ± 0.1	19.9 ± 2.4^∗∗∗^	2.7 ± 0.3^$$$^	2.2 ± 0.3^$$$^	6.1 ± 1.1^∗^^$$$^
Cholesterol (mg/mg of liver)	7.8 ± 0.7	32.7 ± 9.9^∗∗∗^	10.6 ± 1.6^$$^	13.9 ± 1.6^$^	14.2 ± 2.5^$^
Glycogen storage (mg/mg of liver)	0.0417 ± 0.0034	0.02405 ± 0.0027^∗^	0.04615 ± 0.0071^$^	0.0335 ± 0.001	0.0446 ± 0.0084^$^
Plasma *γ*GT (U/L)	17.3 ± 0.58	34.1 ± 11.08^∗^	25.9 ± 0.35	27.2 ± 0.69^∗^	28.78 ± 2.85^∗^

## Data Availability

The data used to support the findings of this study are available from the corresponding author upon request.

## Abbreviations

ABTS: 2,2′-Azino-bis-(3)-ethylbenzthioazoline-6-sulfonic acid
AMPK: AMP-activated protein kinase
AUC: Area under the curve
ChREBP: Carbohydrate-responsive element-binding protein
DHE: Dihydroethidine
eNOS: Endothelial nitric oxide synthase
ERK: Extracellular signal-regulated kinases
FFA: Free fatty acids
GS: Glycogen synthase
F&V: Fruits and vegetables
HFHF: High-fat high-fructose diet
HFHF/HFHFRC: Rats who received 2 months of HFHF and shift to 2 months of HFHF enriched on RC
HFHF/ND: Rats who received 2 months of HFHF and shift to 2 months of ND
HFHF/NDRC: Rats who received 2 months of HFHF and shift to 2 months of ND enriched on RC
HOMA2-IR: Homeostatic model assessment indexes of insulin resistance
HPLC: High-performance liquid chromatography
ipGTT: Intraperitoneal glucose tolerance test
IRS1: Insulin receptor substrate 1
MAPK: Mitogen-activated protein kinases
MEK: MAPK/ERK kinase
NADPH oxidase: Nicotinamide adenine dinucleotide phosphate oxidase
NAFLD: Nonalcoholic fatty liver disease
NASH: Nonalcoholic steatohepatitis
ND: Normal diet
NF-*κ*B: Nuclear factor-kappa B
NO: Nitric oxide
Nrf2: Nuclear factor erythroid-2-related factor 2
OS: Oxidative stress
PPAR: Peroxisome proliferator-activated receptors
RC: Red cabbage
ROS: Reactive oxygen species
SDS: Sodium dodecyl sulfate
SOD: Superoxide dismutase
SREBP1 and 2: Sterol regulatory element-binding proteins 1 and 2
SIRT1: Sirtuin 1
TAOC: Total antioxidant capacity
TBARS: Thiobarbituric acid reactive substances
TG: Triglycerides
T2D: Type 2 diabetes.

## Supplementary Materials

Supplementary Materials Table S1: food composition and consumption. Figure S1: HPLC-AOx profile of the sugar-free polar extract of RC uncooked (A) and cooked (B). Upper chromatogram: 280 nm. UV-absorbance; lower chromatogram: detection of the ABTS ^+^radical cation decolorization at 412 nm after post column reaction. (C) Graphical representation of the summing peak TEAC values.
