# Supplementary material for: Treatment of NASH with Antioxidant Therapy: Beneficial Effect of Red Cabbage on Type 2 Diabetic Rats
Source: Oxid Med Cell Longev. 2018 Sep 30;2018:7019573. doi: 10.1155/2018/7019573 (PMC6186311; doi:10.1155/2018/7019573)

Supplemental information

Experimental procedures

Evaluation of anti-oxidant capacity of individual molecules

Compound separation, as previously described(1,2), was operated by High Performance Liquid Chromatography (HPLC) coupled with an on-line antioxidant capacity detection system. The system consist in a Dionex Ultimate Autosampler WPS3000FC, Dual Gradient Pump 3600 SD, Diode Array Detector 3000 (RS), and a Variable Wavelength Detector 3100. The chromatographic column was an ACE C18-PFP (Advanced Chromatography Technologies LTD, Scotland, 250x 4.6 mm; 5 mm). A binary solvent system was used at a flow rate of 1 mL/min with A: Milli-Q water/trifluoroacetic acid (99.9/ 0.1) (v/v) and B: acetonitril, applying the gradient of 0.5 mL/min. The mixture was guided through a 25-m long PTFE reaction coil with 0.25 mm internal diameter to a second UV detector, where $ABTS^{\cdot+}$ decolourisation was monitored at 412 nm with the 3100 variable wavelength detector. The geometry of the coil and the flows driven by the two pumps offer a reaction time of 0.81 min before the second detection. The presence of the separated molecules was detected by visualising positive peaks with the DAD detector. Their anti-oxidant capacity was determined and quantified when a corresponding negative peak was detected by the second detector, using an external Trolox calibration curve. Results were processed with the Dionex Chromeleon 6.8 software. The negative chromatograms have been aligned with the positive chromatograms, with an offset of e 0.81 min.

References

1. Van der Werf R DS, Le Grandois J, Aoudé-Werner D, Digel F, Ennahar S, Sigrist S, Marchioni E. Determination of active radical scavenging compounds in polar fruit and vegetable extracts by an on-line HPLC method. *LWT - Food Science and Technology* 62: 152-159, 2015.

2. Van der Werf R. SS, Ros S., Aoude-Werner D., Le Grandois J., Ennahar S., Digel F., Marchioni E. Chromatographic on-line detection of bioactives in food. *Functional Foods in Health and Disease* 3: 323-331, 2013.

Table and figures legends

Table S1: Food composition and consumption

Variables	ND	HFHF	HFHF/ND	HFHF/NDRC	HFHF/HFHFRC
**Food composition**					
Energy (kcal/100g)	287	456	287	281.23	433.06
Proteins (g/100g)	16.1	17.5	16.1	15.43	16.69
Lipids (g/100g)	3.1	21.4	3.1	2.79	19.26
Carbohydrates (g/100g)	46.7	46.5	46.7	45.80	45.62
Fibers (g/100g)	3.9	3.5	3.9	5.59	5.23
**Food and beverage consumption**					
Food intake (g/d)	25.10±0.70	**15.25±0.72*** **	**20.57±1.70***$$$**	**20.30±1.92***$$$**	**12.38±0.56***$$**
Beverage intake (mL/d)	40.9±3.1	**70.2±14.0***	37.1±1.5$$	27.7±4.1$$$	**75.9±6.03***
Total calorie intake (kCal/d)	72.04±2.01	76.57±3.88	**59.03±5.22**$$**	**45.02±4.37***$$$**	**61.21±2.59***$$$**

Foods composition and food and beverage consumption after 4 months of normal diet (ND), high-fat/high-fructose diet (HFHF), HFHF 2 months+ND 2 months (HFHF/ND), HFHF 2 months+ND with RC 2 months (HFHF/NDRC), and HFHF 2 months+HFHF with RC 2 months (HFHF/HFHFRC). Results obtained in six experiments are presented. * represent significant results *vs.* ND; $ *vs.* HFHF; # *vs.* HFHFND.

Figure S6: HPLC-AOx profile of the sugar free polar extract of RC uncooked (A) and cooked (B). Upper chromatogram: 280 nm UV-absorbance; lower chromatogram: detection of the $ABTS^{\cdot +}$ radical cation decolorization at 412 nm after post-column reaction. (**C**) Graphical representation of the summing peak TEAC values.

A.

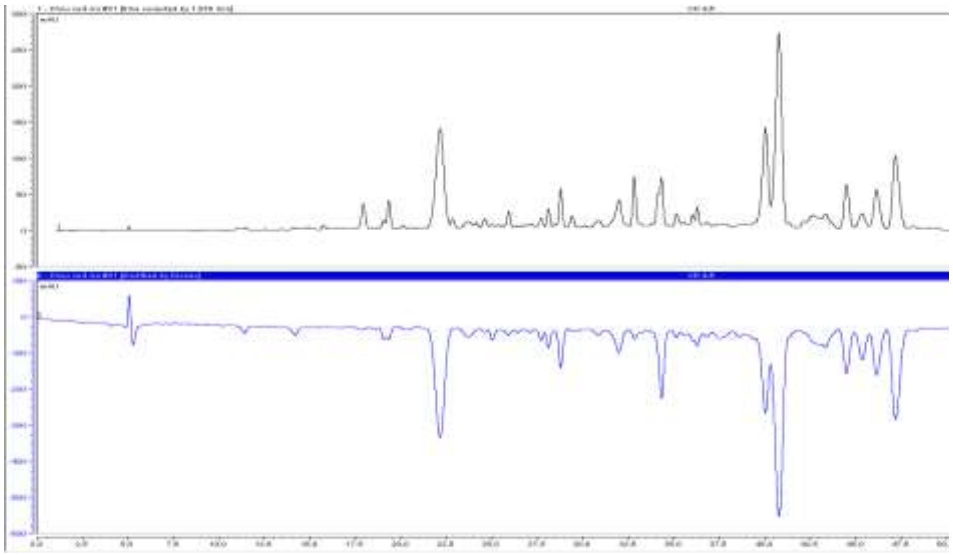

C.

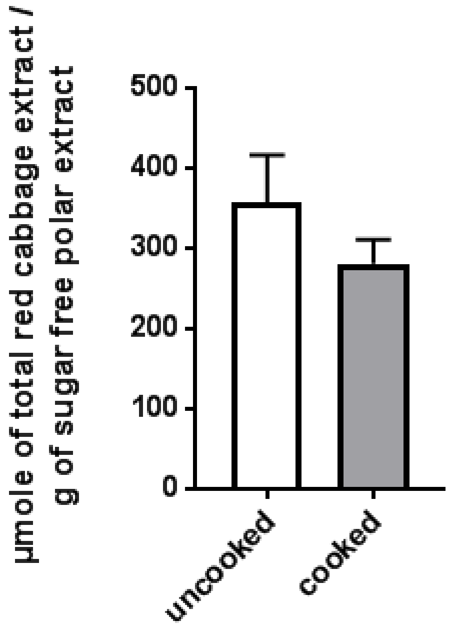

B.

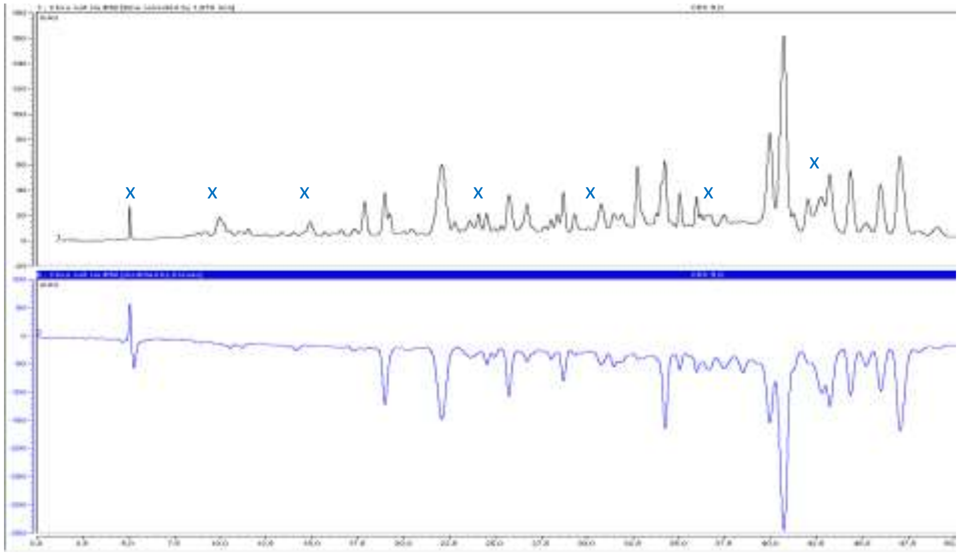

Supplement: Supplementary Materials — Table S1: food composition and consumption. Figure S1: HPLC-AOx profile of the sugar-free polar extract of RC uncooked (A) and cooked (B). Upper chromatogram: 280 nm. UV-absorbance; lower chromatogram: detection of the ABTS +radical cation decolorization at 412 nm after post column reaction. (C) Graphical representation of the summing peak TEAC values. [file 7019573.f1.pdf]
